# Effects of sterilization treatments on bulk and surface properties of nanocomposite biomaterials

**DOI:** 10.1002/jbm.b.32928

**Published:** 2013-09-11

**Authors:** Maqsood Ahmed, Geoffrey Punshon, Arnold Darbyshire, Alexander M Seifalian

**Affiliations:** 1 Centre for Nanotechnology, Biomaterials & Tissue Engineering, Division of Surgery & Interventional Science, University College LondonLondon, UK; 2 Centre of Mathematics and Physics in the Life Sciences and Experimental Biology (CoMPLEX))London, UK; 3 Royal Free Hampstead NHS Trust HospitalLondon, UK

**Keywords:** sterilization, Biomaterials, polymer degradation, polyurethane

## Abstract

With the continuous and expanding use of implantable biomaterials in a clinical setting, this study aims to elucidate the influence of sterilization techniques on the material surface and bulk properties of two polyurethane nanocomposite biomaterials. Both solid samples and porous membranes of nondegradable polyhedral oligomeric silsesquioxane poly(carbonate-urea) urethane (POSS-PCU) and a biodegradable poly(caprolactone-urea) urethane (POSS-PCL) were examined. Sterilization techniques included conventional steam sterilization (autoclaving), gamma irradiation, and disinfection via incubating with ethanol (EtOH) for 10 min or 24 h. After treatment, the samples were examined using gel permeation chromatography (GPC), attenuated total reflectance Fourier transform infrared spectroscopy, and tensiometry. Cytotoxicity was evaluated through the culture of endothelial progenitor cells and the efficacy of sterilization method was determined by incubating each sample in tryptone soya broth and fluid thioglycollate medium for cultivation of microorganisms. Although EtOH did not affect the material properties in any form, the samples were found to be nonsterile with microbial growth detected on each of the samples. Gamma irradiation was not only effective in sterilizing both POSS-PCU and POSS-PCL but also led to minor material degradation and displayed a cytotoxic effect on the cultured cells. Autoclaving was found to be the optimal sterilization technique for both solid and porous membranes of the nondegradable POSS-PCU samples as it was successful in sterilizing the samples, displayed no cytotoxic side effects and did not degrade the material. However, the biodegradable POSS-PCL was not able to withstand the harsh environment during autoclaving, resulting in it losing all structural integrity.

## Introduction

Biomaterials play a critical role in the healthcare industry with applications ranging from medical implants, prosthetics, and diagnostic devices. However, the ever increasing use of implantable medical devices has led to a significant increase in the number of infections, with at least half of all cases of nosocomial infections associated with medical devices.^1^ For example, up to 6% of prosthetic grafts can encounter difficulties with infection resulting in approximately $640m in healthcare costs.^2^ In addition, there are significant associated morbidity and mortality rates with amputation rates approaching 11% and re-infection found in 18% of patients. In 17–40% of patients with infected grafts it can lead to death. The most effective method to minimise the risk of infection is to use sterile materials and aseptic techniques. Direct implantation of device with microorganisms attached is a common and avoidable method in which infection can occur necessitating the need for effective sterilization methods.^3^

Sterilization can be achieved in a number of ways, including steam and gamma irradiation. Although numerous investigations have characterized microbial activity after sterilization, as a marker of efficacy, the impact of sterilization technique on the material bulk, and surface properties is often ignored, whereas it is often the material bulk and surface properties that determine the success of the implant/device. The sterilization process usually involves physical or chemical treatment, which results in the elimination of organic macromolecules and/or microorganisms. Given the nature of their action, the techniques can also react with the biomaterial. Steam sterilization can lead to hydrolysis, softening, and degradation of the polymer because of the high temperature, pressure, and humidity.^4^ Gamma irradiation is known to cause chain scission and cross-linking, which can adversely affect material properties.^5^ Furthermore, both steam and gamma have been known to deform and yellow the polymeric materials. Immersing in ethanol is a milder technique used *in vitro* to disinfect the polymer sample. It is particularly useful for disinfecting biodegradable tissue engineering scaffolds, such as glycolic acid-based materials, which, by their very nature, tend to be fragile.^6^

Polyurethanes (PUs) represent a large family of biomaterials commonly found in a clinical setting. They consist of different organic units linked together by urethane bonds [[—]NHC(O)O[—]], which affords them a high degree of versatility. With excellent biocompatibility and mechanical properties, PUs are amenable to a number of applications, including catheters, biomedical implants, and tissue engineering scaffolds.^7^ However, chronic *in vivo* failure observed on prolonged implantation, primarily because of polymer degradation, is a major stumbling block for their continued use.^8^ The polyester-based soft segments of PUs are prone to hydrolysis, whereas polyether-based PUs are susceptible to oxidative attack.

**Figure 1:**
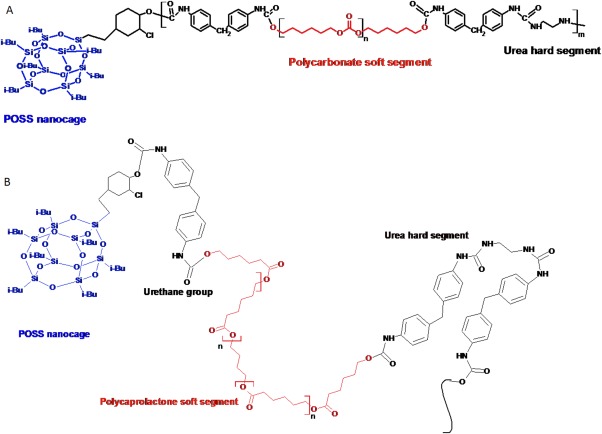
Molecular structures of (A) POSS-PCU and (B) POSS-PCL synthesized investigated in this study. [Color figure can be viewed in the online issue, which is available at wileyonlinelibrary.com.]

In an effort to produce more durable and biostable PUs, our group tethered polyhedral oligomeric silsesquioxane (POSS) nanocages to a poly(carbonate-urea) urethane (PCU) backbone (Figure 1). Siloxanes are well known to be biostable and resistant to oxidation and hydrolysis because of the strong intermolecular forces between the constituent molecules and a strong framework with shorter bond lengths. However, siloxanes are mechanically fragile exhibiting particularly poor tear strengths, which make siloxane-based polymers unsuitable for many applications. By covalently attaching the POSS moiety to the polymer network, our group was able to exploit the beneficial effects of improved biostability afforded by the POSS molecule, without compromising the mechanical integrity of the PU. The polyhedral oligomeric silsesquioxane-poly(carbonate-urea) urethane (POSS-PCU) produced was found to be more resistant to degradation *in vitro* and *in vivo*, possess antithrombogenic properties, and sustain cell growth on its surface; a combination of properties making it suitable for a number of biomedical applications, including heart valves, prosthetic grafts, and stents.^9^–^12^ In conjunction with POSS-PCU, a POSS-modified biodegradable aliphatic caprolactone polyurethane, poly(caprolactone-urea) urethane (POSS-PCL), was also developed for tissue engineering applications.^13^ The POSS-based materials developed in our laboratory are beginning to find clinical use, with the most recent example being a porous synthetic scaffold for use in a tracheobronchial transplant.^14^

The purpose of this study was to evaluate the impact of the sterilization technique on the bulk and surface properties of solid samples, referred to as cast sheets, and porous membranes of POSS-PCU and POSS-PCL polymers developed in our laboratory. Porous membranes were fabricated using a phase separation method described previously.^9^ The treatments include 70% ethanol, steam, and gamma irradiation. Changes to the molecular weight, mechanical strength, surface chemistry, and cytotoxicity were evaluated and compared with an unsterilized control. The efficacy of the sterilization method was also determined.

## Materials and Methods

### Polymer synthesis

#### POSS-PCU

Polycarbonate polyol, 2000 mwt (Bayer Material Science GmbH) and trans-cyclohexanechloroydrinisobutyl-silsesquioxane (Hybrid Plastics Inc) were placed in a 250-mL reaction flask equipped with mechanical stirrer and nitrogen inlet. The mixture was heated to 135°C to dissolve the POSS cage into the polyol and then cooled to 70°C. Flake 4,4′-methylenebis (phenyl isocyanate) (MDI) were added to the polyol blend and then reacted, under nitrogen, at 75–85°C for 90 min to form a pre-polymer. Dimethylacetamide (DMAc) was added slowly to the pre-polymer to form a solution; the solution was cooled to 40°C. Chain extension of the pre-polymer was performed by the drop wise addition of a mixture of ethylenediamine and diethylamine in DMAc to form a solution of POSS-modified polycarbonate urea-urethane in DMAc.

#### POSS-PCL

Dry polycaprolactone diol (2000 mwt) and POSS were placed in a 250-mL reaction flask equipped with mechanical stirrer and gas inlet. The mixture was heated to 135°C to dissolve the POSS cage into the polyol and then cooled to 90°C. 4, 4′ methylenebis(cyclohexyl isocyanate) (Desmodur W, Bayer) was added to the polyol blend and then reacted, under nitrogen, at 90°C for 120 min with catalyst (Bismuth neodecanoate) to form a pre-polymer. Dry DMAc was added slowly to the pre-polymer to form a solution; the solution was cooled to 40°C. Chain extension of the pre-polymer was performed by the drop wise addition of a mixture of ethylenediamine and diethylamine in dry DMAc. After completion of the chain extension, 1-butanol in DMAc was added to the polymer solution forming a POSS-PCL solution.

Unless otherwise stated, all chemicals and reagents were purchased from Aldrich Limited, Gillingham, UK.

### Sample preparation

#### Cast sample

The 18% (w/w) solutions of polymer in DMAc were cast onto glass petri dishes and left in an oven at 60°C overnight to evaporate the solvent. The resulting solid sheets of polymer were then sterilized accordingly and used for future experiments.

#### Porous sample

Sodium bicarbonate [NaHCO_3_, 50% (w/w), 40 µm particle size; Bruner Mond, Cheshire, UK] was dispersed into an 18% (w/w) solution of the polymers in DMAc containing 2% Tween 80 surfactant. The mixture was mixed and degassed in one process using a Thinky ARE 250 mixer (Intertonics, Oxfordshire, UK) resulting in a viscous slurry, which was then cast onto a stainless steel sheet and placed in distilled water for 48 h at room temperature. A sheet of porous polymer was formed via the immersion precipitation process as a result of solvent exchange with distilled water. The polymer sheets were left immersed in distilled water for a period of 48 h to ensure complete removal of solvent and NaHCO_3_. The membranes were then removed from the steel support, air dried for a further 48 h, and sterilized appropriately for future experiments.

### Sterilization

#### Gamma irradiation

Samples were packed in sterilization pouches and irradiated with a dose of 28.4 kGy at room temperature, using a 60Co gamma-ray source (Isotron, Berkshire, UK). Samples were exposed to the source on a continuous path for a period of 10 h.

#### Autoclave

Autoclaving involves exposing the biomaterials to saturated steam at 121°C for a minimum of 15 min at pressures of 115 kPa. The samples were autoclaved and then left overnight for cooling.

#### Ethanol

Polymer discs were incubated with 70% (v/v) ethanol and left in a roller mixer for 10 min or 24 h. Samples were then washed (5×) in distilled water and stored for future use.

### Material characterization

#### Tensiometry

Samples were cut longitudinally into a dogbone-shaped specimen, 20 × 4 mm, using a sharp cutter and mechanical press ensuring a clean cut with no flaws or stress aggregation. However, because of the random nature of pore size and porosity in the porous samples, variability is to be expected. The thickness of the samples was determined using an electronic micrometer. Stress–strain profiles were characterized using a uniaxial load testing machine (Instron 5565, UK) and the Young’s modulus, ultimate tensile strength (UTS), and elongation at break obtained (*n* = 6).

#### Attenuated total reflectance Fourier transform infrared spectroscopy

The chemical structure of the PU samples, after exposure to the various methods of sterilization, was evaluated via attenuated total reflectance Fourier transform infrared (ATR-FTIR) spectroscopy (JASCO FT/IR 4200). Thirty scans were taken for each sample between 600 and 4000 cm^−1^ (*n* = 6).

#### Gel permeation chromatography

Solutions of each sample were prepared by adding 15 mL of dimethylformamide (DMF) to 30 mg of sample and left to dissolve on a roller mixer over night. The samples were analyzed using a PL-GPC 50 system (Agilent Technologies) equipped with PLGel column guard and 3 PLGel 5 µm mixed bed-C columns (300 × 7.5 mm). The measurement was performed at 50°C in DMF, and the eluent was pumped at the constant flow rate of 1.0 mL/min. The system was calibrated by performing Universal Calibration with PL- polystyrene standard and a set of PL-EasyVial PS-H polystyrene standards of known molecular weights (a 12-point calibration curve in the nominal range of 162–6,000,000 g/mol). The detection was done using a PL-BV 400RT viscometer and a PL-RI differential refractometer. The data were collected and analyzed using Varian “Cirrus Multi detector” software (*n* = 3). Results are presented as percentages of untreated control.

### Cytotoxicity

#### Endothelial progenitor cell extraction

Blood samples were collected after consent from healthy adult human volunteers. Twenty-four milliliter samples were collected by venepuncture in EDTA blood tubes (Sarstedt, U.K.). After collection, samples were mixed and used for cell isolation within 1 h of collection.

The mononuclear fraction of the blood was isolated using Histopaque 1077 (Sigma-Aldrich, U.K.). In brief, 3 mL of Histopaque 1077 was added to each of eight 12 mL polystyrene centrifuge tubes (Falcon, UK). Three milliliter of blood was carefully layered on top. The tubes were then centrifuged at 400*g* for 30 min at room temperature. The mononuclear fraction was separated from each tube, and the samples pooled in 30 mL universal tubes (Falcon, U.K.). Ten milliliter of Hank’s balanced salt solution (HBSS; Invitrogen, UK) was then added slowly to each tube and the contents mixed. The tubes were centrifuged at 250*g* for 10 min at room temperature. The cells were washed twice by removing the supernatant, resuspending in 10 mL HBSS, and centrifuging at 250*g* for 10 min at room temperature. Finally, the isolated cells were resuspended in 5 mL cell culture medium (CCM): M199 supplemented with 20% fetal bovine serum, and penicillin/streptomycin (Invitrogen). Cells were then counted using a hemocytometer and seeded onto polymer samples as below.

#### Endothelial progenitor cell culture

Cells were seeded onto polymer discs (*n* = 4) in a 24 well plate (Falcon, UK) at a seeding density of 5 × 10^5^ cells/well in 1 mL CCM Cells were then cultured for 7 days.

#### Alamar Blue assay

Cell metabolism was assessed by Alamar blue™ (AB) assay at day 7. In brief, medium was removed from the wells and 1 ml 10% AB in CCM added. After a 4-h incubation, samples of AB/CCM were removed and measured on a Fluroskan Ascent FL (Thermo Labsystems, UK) fluorescent plate reader (excitation 530 nm, emission at 620 nm).

### Efficacy of sterilization

All samples were tested for the effectiveness of sterilization. Samples were immersed in tryptone soya broth (TSB) and fluid thioglycollate medium (THY) for cultivation of microorganisms (Wickham Laboratories, Hampshire) for a period of 14 days at temperatures of 20–25°C for TSB and 30–35°C for THY. Sterile broth was used a negative control and unsterilized samples as positive controls. The broths were examined macroscopically every 1–3 days with clouding of the broth indicative of contamination and inefficient sterilization, whereas a clear broth would indicate no infection and an efficient sterilization of the samples (*n* = 3).

## Results

### Visual inspection

All samples withstood treatment with EtOH well, irrespective of incubation time. Although the POSS-PCU samples were unaffected by the autoclaving process, the POSS-PCL samples were destroyed; therefore, it was not possible to examine the autoclaved POSS-PCL samples further. Both the POSS-PCU and POSS-PCL samples held up well against gamma irradiation with slight discolouring/yellowing of the POSS-PCU samples being observed.

### Mechanical test

**Figure 2:**
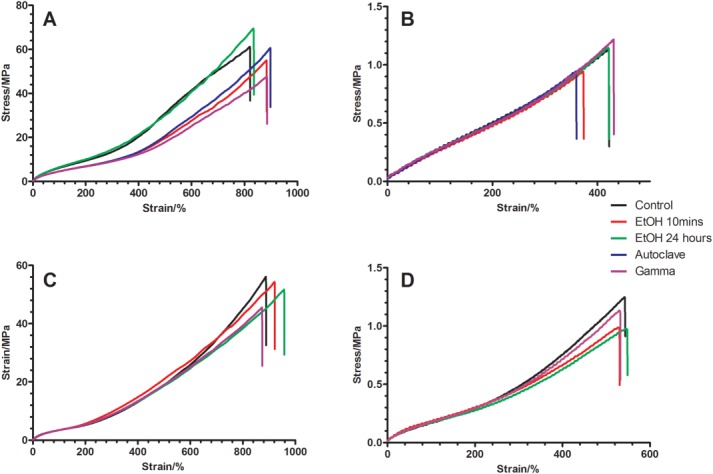
Typical stress–strain profile of (A) cast POSS-PCU, (B) porous POSS-PCU, (C) cast POSS-PCL, and (D) porous POSS-PCL nanocomposites after sterilization with EtOH (10 min and 24 h), autoclave, and gamma irradiation. Curves are typical of those expected for an elastomeric polyurethane sample. The ultimate tensile strength, Young’s modulus, and elongation at break values are provided in Table 1. [Color figure can be viewed in the online issue, which is available at wileyonlinelibrary.com.]

Figure 2 shows the stress–strain curves of the materials after exposure to EtOH, autoclaving, and gamma irradiation, with quantitative values presented in Table 1.

**Table 1:** Mechanical Properties (Young’s Modulus, Ultimate Tensile Strength, and Elongation at Break) of POSS-PCU and POSS-PCL Samples After Sterilization via a Number of Different Techniques (*n* = 6)

Sample		Sterilization Technique	Young’s Modulus (MPa)	Tensile Strength (Mpa)	Elongation at Break (%)
POSS PCU	Cast	Control	8.61 ± 0.81	62.35 ± 6.71	823.96 ± 28.03
		EtOH 10 min	7.75 ± 0.47	56.48 ± 2.98	875.36 ± 47.01
		EtOH 24 h	7.75 ± 0.64	66.67 ± 3.73	828.59 ± 29.09
		Autoclave	9.16 ± 0.62	62.39 ± 5.72	904.86 ± 37.40^a^
		Gamma	6.49 ± 0.46^a^	48.87± 5.04^a^	853.24 ± 51.02^a^
	Porous	Control	0.35 ± 0.02	1.08 ± 0.08	440.76 ± 11.66
		EtOH 10 min	0.32 ± 0.03	0.98 ± 0.06	449.33 ± 25.45
		EtOH 24 h	0.32 ± 0.04	1.07 ± 0.07	437.51 ± 31.43
		Autoclave	0.33 ± 0.04	0.97 ± 0.05	382.84 ± 35.46^a^
		Gamma	0.35 ± 0.03^a^	1.22 ± 0.05^a^	407.03 ± 9.87^a^
POSS PCL	Cast	Control	6.91 ± 0.93	57.71 ± 2.32	885.73 ± 85.00
		EtOH 10 min	5.61 ± 0.82	55.05 ± 2.42	938.88 ± 54.99
		EtOH 24 h	5.92 ± 0.60	49.55 ± 4.18	968.32 ± 40.45
		Autoclave	n/a	n/a	n/a
		Gamma	5.03 ± 0.278^a^	42.89 ± 4.82^a^	865.69 ± 114.47
	Porous	Control	0.28 ± 0.04	1.36 ± 0.13	593.76 ± 38.85
		EtOH 10 min	0.22 ± 0.03	1.04 ± 0.11	581.87 ± 29.58
		EtOH 24 h	0.20 ± 0.01	0.97 ± 0.04	577.52 ± 12.42
		Autoclave	n/a	n/a	n/a
		Gamma	0.23 ± 0.02	1.03 ± 0.07	581.74 ± 31.31

a p < 0.05

Both POSS-PCU and POSS-PCL tolerated ethanol treatment well—both cast sheets and porous samples. No significant difference was seen between the Young’s modulus, UTS, or elongation at break in any of the samples compared with the untreated control.

Similarly, autoclaving did not affect the Young’s modulus or UTS of the cast samples of POSS-PCU. However, there was a slight significant (*p* < 0.05) increase in the elongation at break compared with the control, whereas autoclaving the porous samples resulted in a minor decrease in elongation at break.

On gamma irradiation, the porous samples withstood the effects of gamma irradiation well; however, the cast samples suffered a significant (*p* < 0.001) reduction in mechanical properties. The UTS for POSS-PCU decreased from 62.3 ± 6.7 MPa to 48.9 ± 5.0 MPa, whereas POSS-PCL went from 57.7 ± 2.3 MPa to 42.9 ± 4.8 MPa (*p* < 0.001). The reduction in UTS translated itself to the Young’s moduli, which were both significantly reduced for cast samples of POSS-PCU and POSS-PCL.

### ATR-FTIR

**Figure 3:**
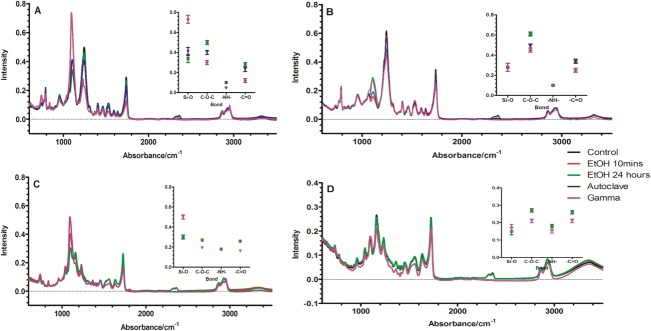
ATR-FTIR analysis of (A) cast POSS-PCU, (B) porous POSS-PCU, (C) cast POSS-PCL, and (D) porous POSS-PCL nanocomposites after sterilization with EtOH (10 min and 24 h), autoclave, and gamma irradiation. A summary of the changes in key peak intensities are inset (*n* = 6, *p* < 0.05). [Color figure can be viewed in the online issue, which is available at wileyonlinelibrary.com.]

ATR-FTIR spectroscopy was used to analyse surface chemical changes on sterilization with the results summarized in Figure 3. The peak assignment was as follows: 1100 cm^−1^ (Si-O-Si), 1240 cm^−1^ (urethane C[—]O[—]C), 1400 cm^−1^ (C[—]C aromatic ring), 1540 cm^−1^ (N[—]H and C[ ]N), 1589 cm^−1^ (C[ ]C aromatic), 1632 cm^−1^ (NH_2_), 1736 cm^−1^ (C[ ]O), 3422 cm^−1^ (N[—]H).

Ethanol treatment had no significant impact on either of the POSS-PCU or POSS-PCL samples, neither cast nor porous. A slight increase in peak at 1100 cm^−1^, attributed to the POSS moiety was detected for the porous sample incubated in EtOH for 24 h. Meanwhile, autoclaving POSS-PCU caused a slight decrease (*p* < 0.05) in the intensity of the ether peak at 1245 cm^−1^ for both the cast and porous samples.

Exposure of POSS-PCU to gamma irradiation led to significant reductions in peak intensity at 1245, 1540, and 1740 cm^−1^ on both cast and porous samples. There was, however, an increase in the intensity of the peak at 1095 cm^−1^ for the cast POSS-PCU sample (*p* < 0.001). Similarly, both POSS-PCL samples suffered a reduction in peak intensity, after exposure to gamma irradiation, at 1730, 1540 1240, and 1165 cm^−1^. However, the cast sample of POSS-PCL also exhibited an increase in peak intensity at 1100 cm^−1^.

### Gel permeation chromatography

**Figure 4:**
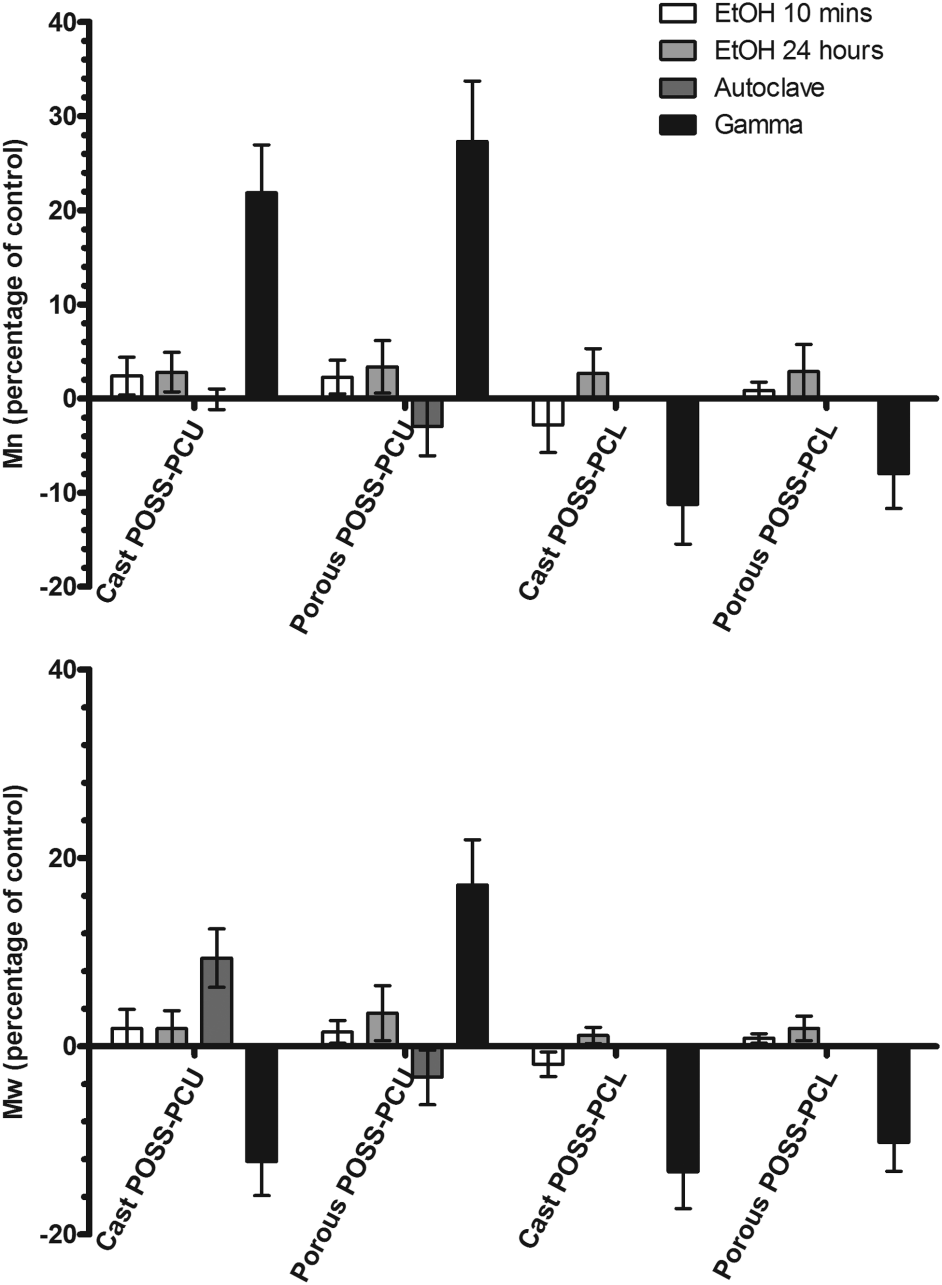
The percentage change (compared with unsterilized control) in number average (*M*_n_, top) and weight average (*M*_w_, bottom) molecular weight of POSS-PCU and POSS-PCL, both cast and porous samples after sterilization via a 10-min incubation in EtOH, 24 h incubation in EtOH, autoclaving, and gamma irradiation (mean ± SD, *n* = 3). The molecular weights of the unsterilized controls were as follows: POSS-PCU (*M*_w_ = 108 500, *M*_n_ = 42,600), POSS-PCL (62 800, *M*_n_ = 31,200).

Gel permeation chromatography (GPC) results are summarized in Figure 4. The untreated POSS-PCU was found to have a weight average molecular weight (*M*_w_) of 108,500 and a number average molecular weight (*M*_n_) of 42,600, whereas POSS-PCL had an *M*_w_ of 62,800 and *M*_n_ of 31,200. After treatment with EtOH, there was a negligible impact on either *M*_n_ or *M*_w_ regardless of incubation time, for any of the four samples.

Autoclaving the cast sheet of POSS-PCU resulted in a 9.3% increase (*p* < 0.05) in *M*_w_ but no change in *M*_n_ possibly suggesting a degree of cross linking. No changes in molecular weight distributions were detected in autoclaved porous samples of POSS-PCU.

Exposure to gamma irradiation had a significant impact on all of the samples. The *M*_n_ of cast POSS-PCU increased significantly by 21.9%, whereas the *M*_w_ decreased by 12.3% (*p* < 0.001). Meanwhile, gamma irradiation increased both the *M*_n_ and *M*_w_ of the porous POSS-PCU by 27.3 and 17.1%, respectively. Both cast and porous samples of POSS-PCL exhibited a significant (*p* < 0.001) decrease in *M*_n_ (11.2 and 7.2%) and *M*_w_ (13.4 and 10.4%), respectively, after exposure to gamma irradiation.

### Cytotoxicity

**Figure 5:**
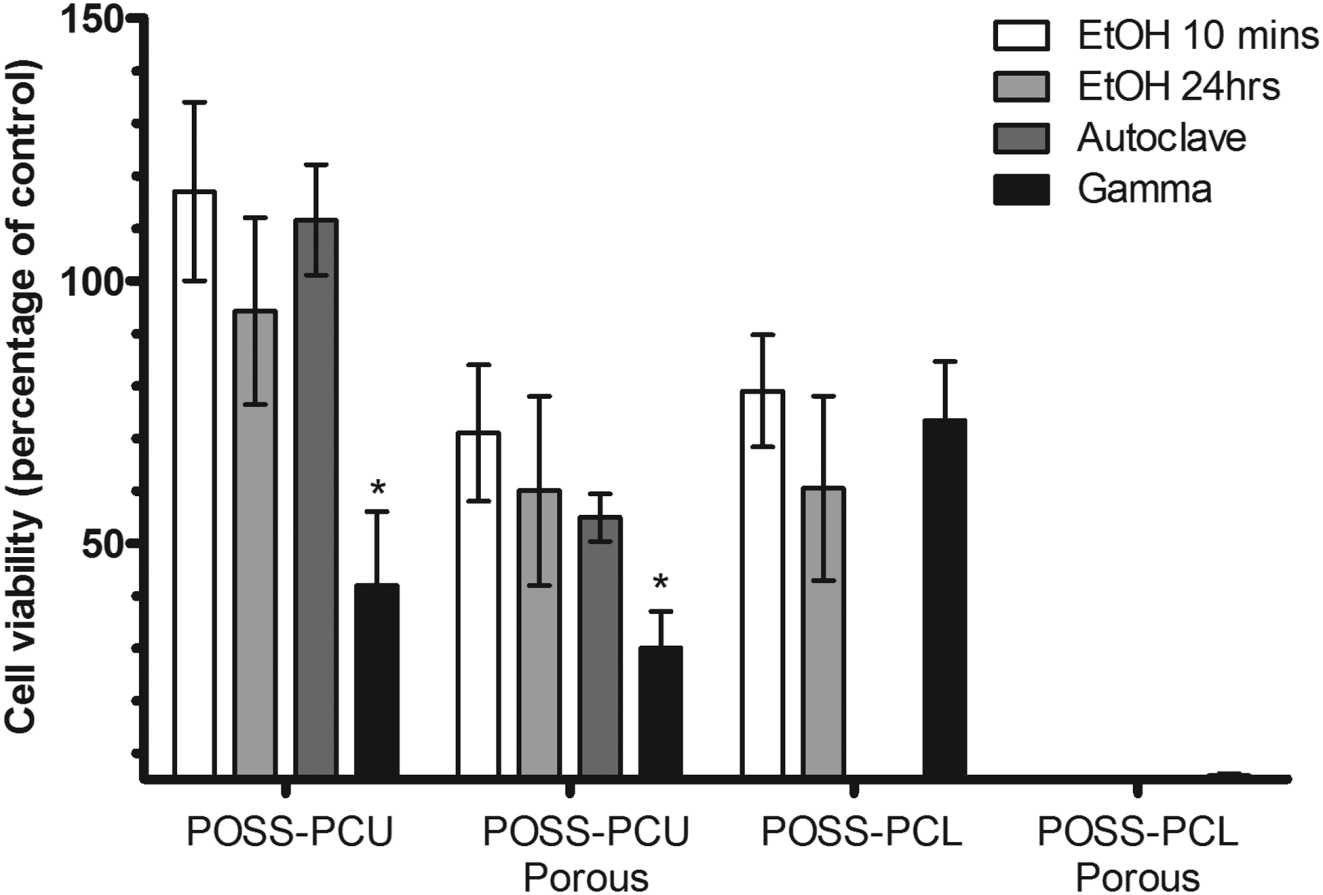
Cell viability after 7 days of incubation, as determined by Alamar blue assay. Results (mean ± SD) are presented as a percentage of the control cells grown on tissue culture plastic (*n* = 4, *p < 0.001). Gamma irradiation seemed to reduce the number of viable cells by approximately 50% on cast POSS-PCU and 75% on the porous samples of POSS-PCU, whereas no significant differences were observed in cell growth on cast POSS-PCL samples. No cell growth was seen on the porous samples of POSS-PCL.

The results of the Alamar Blue assay are presented in Figure 5 as a ratio of viable cells compared with control cells cultured on the tissue culture plastic, after 7 days of incubation. Although no significant differences were detected between cell viability on EtOH-treated and autoclaved samples, sterilizing via gamma irradiation reduced cell viability by approximately 50%, compared with EtOH-treated and autoclaved samples, on both cast and porous POSS-PCU samples. Sterilization technique had no significant impact on cell growth on the cast samples of POSS-PCL and no cell growth was observed on the porous POSS-PCL samples.

### Efficacy of sterilization

The polymeric materials were incubated in TSB and THY to test the efficiency of sterilization with the resultant level of bacterial growth reported in Table 2. THY is a viscous growth medium with reduced oxygen levels, which tests the growth of anaerobic bacteria and other organisms capable of growing in reduced oxygen tension. No evidence of bacterial growth was observed on any of the materials tested after incubation in THY. TSB, however, is a general growth media for aerobic microorganisms and is designed for the growth of aerobic bacteria and yeasts and moulds. The materials sterilized via EtOH incubation were not fully sterile and sustained the growth of bacteria on all three samples tested of each material. Autoclaving and gamma irradiation appear to be far more efficient sterilization techniques as no evidence of bacterial growth was observed on any of the polymeric materials.

**Table 2:** Bacterial Growth Observed on Each Sample After Incubation *in Tryptone* Soya Broth *(TSB) and Fluid Thioglycollate* Medium *(THY) for* Cultivation *of* Microorganisms

			Growth in Media	
Sample		Sterilization Technique	TSB	THY
POSS PCU	Cast	EtOH 10 min	3/3	0/3
		EtOH 24 h	2/3	0/3
		Autoclave	0/3	0/3
		Gamma	0/3	0/3
	Porous	EtOH 10 min	2/3	0/3
		EtOH 24 h	3/3	0/3
		Autoclave	0/3	0/3
		Gamma	0/3	0/3
POSS PCL	Cast	EtOH 10 min	3/3	0/3
		EtOH 24 h	3/3	0/3
		Autoclave	n/a	n/a
		Gamma	0/3	0/3
	Porous	EtOH 10 min	3/3	0/3
		EtOH 24 h	2/3	0/3
		Autoclave	n/a	n/a
		Gamma	0/3	0/3

*Samples were tested in triplicates, and no evidence of bacterial growth was found on any of the materials tested* that *were cultivated in THY. Growth of bacteria was observed on all three samples of each polymeric material* steriliz*ed via EtOH incubation and cultivated in TSB. Autoclaved and gamma*-*irradiated materials*s seemed *to be fully sterile and did not support any bacterial growth*.

## Discussion

Sterilization is defined as the complete removal or destruction of viable organisms. More practically, however, it is better defined as a process capable of delivering a certain probability that the device is free from viable microorganisms. The physical or chemical agents capable of achieving sterilization without adversely affecting the material quality, function and use, are rather limited for healthcare products. Two of the more common sterilization techniques are moist heat (autoclaving) and ionizing irradiation (gamma irradiation). Additional methods capable of inactivating viable organisms exist, but are less effective than terminal sterilization. These include disinfectants such as EtOH incubation.

Depending on the chemical nature of the material and the processing method, sterilization techniques have been reported to induce changes in material properties. In this study, we investigated the effects of sterilization on two POSS polyurethane nanocomposites: a nondegradable POSS-PCU with an aromatic hard segment and carbonate-based soft segment, and a biodegradable POSS-PCL based on an aliphatic hard segment and caprolactone soft segment. Furthermore, we investigated the effect of sterilization on porous membranes of POSS-PCU and POSS-PCL generated through phase separation. Phase separation is a popular method to create a porous, 3D environment for tissue engineering applications.^15^ The properties of the sterilized samples were compared with untreated controls.

The four samples assessed were incubated with 70% ethanol for a period of 10 min and 24 h. The porous POSS-PCU sample incubated for 24 h in EtOH exhibited a slight increase in the peak at 1100 cm^−1^ possibly indicating a molecular rearrangement after EtOH incubation resulting in the migration of the POSS moiety to the material surface. No further significant changes in ATR-FTIR analysis were found for the EtOH-incubated samples indicated that no changes in the surface chemical composition took place. Furthermore, there were no significant variations in molecular weight or mechanical properties, suggesting that neither surface nor bulk properties of POSS-PCU or POSS-PCL were affected by ethanol irrespective of incubation time. Although ethanol has been shown to be useful in disinfecting lipophilic viruses and Gram-positive, Gram-negative, and acid-fast bacteria; hydrophilic viruses and bacteria spores are resistant to the microbial effects of ethanol making it unsuitable for sterilizing biomedical devices for *in vivo* applications.^16^ Cultivation in TSB broth resulted in microbial growth in all of the samples sterilized with EtOH for 10 min and 24 h. However, no signs of bacterial infection or cytotoxicity were observed in endothelial progenitor cell (EPC) culture with cell growth being in excess of control cells cultured on tissue culture plastic after 7 days of culture for the cast sheets and 75% for the porous samples.

The process of autoclaving requires samples to be exposed to high pressure saturated steam at temperatures of 121°C or more. The combination of high temperature, humidity, and pressure has been shown to be detrimental to PUs with decreases in UTS, hydrolysis, and oxidation being reported in polyether-based PUs.^17^ The caprolactone-based POSS-PCL also could not withstand the effects of autoclaving with extensive deformation of the samples being observed. It is likely that the aliphatic hard segment of the POSS-PCL decrystallizes and loses its structural integrity on exposure to high temperatures. The result of which was that further examination of the autoclaved POSS-PCL samples was not possible.

The nondegradable POSS-PCU samples were, however, relatively unaffected by exposure to autoclaving with the cast and porous samples retaining their shape and structure. GPC analysis suggested there was a minor increase (9.3%) in *M*_w_ of the cast sample, which may explain the slight increase (9.8%) in elongation at break experienced by the autoclaved POSS-PCU. The high temperatures involved in autoclaving could have induced a nominal degree of cross-linking in the sample, which could be responsible for the slight increases in *M*_n_ and elongation at break; however, no evidence was uncovered for cross-linking from the IR spectra.

Although the cast sample displayed a slight increase in elongation at break, the porous sample of POSS-PCU exhibited a small decrease (14.9%) in elongation at break alongside a reduction in ATR-FTIR peak intensity at 1245cm^−1^. These results are consistent with chain scission of the hard segment. The porous samples have a significantly increased surface area, compared with the cast sample, offering a possible explanation for the difference in behavior between the solid sheets and porous samples. Chain scission may be induced by the moist environment at the surface of the material as opposed to the high temperatures being experienced by the bulk. No significant changes were recorded in the molecular weight profile of the porous POSS-PCU sample further reinforcing the belief that chain scission is a surface effect as opposed to bulk. Autoclaving appears to be an effective sterilization technique as no sign of microbial growth was detected in either of the broths. Furthermore, no cytotoxic side effects were noted during cell culture (Figure 4).

Mixed findings have been reported regarding the effects of gamma irradiation on PUs. Although some investigators have found no change in material properties after exposure to gamma irradiation, an equally significant number of publications indicate severe degradation of material.^5^,^17^,^18^ Gamma irradiation is advantageous in that it is a rapid and highly effective sterilization technique: no sign of microbial growth was detected on any of the samples after gamma irradiation (Table 2).

However, gamma irradiation did lead to POSS-PCU samples displaying cytotoxic side effects on the cultured EPCs (Figure 5). Although the exact mechanism behind this observation remains unclear; POSS-PCU has an MDI-based hard segment, and there are reports in the literature of PUs with MDI based hard segments releasing 4,4’-methylene dianiline as a degradation product.^5^,^19^ Aromatic amines are known to be highly cytotoxic and gamma irradiation has been shown to release 4,4′-methylene dianiline previously giving rise to cytotoxic side effects.^20^,^21^

**Figure 6:**
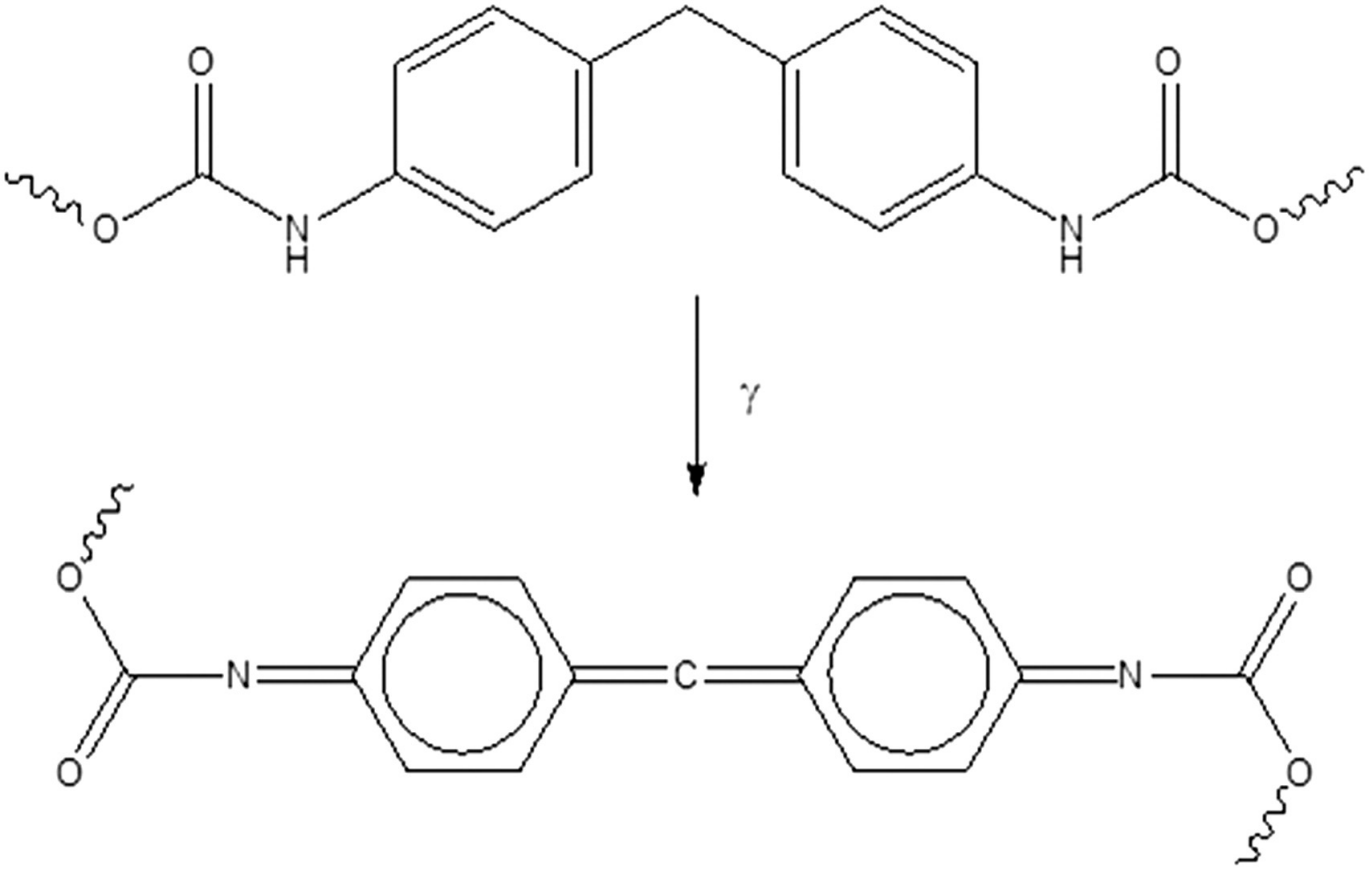
Reaction mechanism for the formation of quinone chromophore responsible for yellowing the POSS-PCU samples.

Gamma irradiation was found to have an impact on both material appearance and properties. Although there were no changes in the appearance of POSS-PCL samples, POSS-PCU had discolored and became yellow. The difference between the two samples is, again, most likely due to the MDI in the aromatic hard segment of POSS-PCU. Exposure to gamma irradiation may have oxidised the central methylene group of the biphenyl leading to a highly conjugated quinone chromophore (Figure 6).

Analysis of the surface functional groups of the cast and porous samples of POSS-PCU via ATR-FTIR provided further evidence of material degradation. Reductions in peak intensities at 1245, 1540, and 1740 cm^−1^ are all consistent with hydrolysis of the hard segment. The reductions in molecular weights for both the cast and porous samples of POSS-PCU would seem to corroborate those findings. However, the *M*_n_ for cast samples of POSS-PCU increased suggesting that a degree of cross linking is also taking place. Because *M*_n_ is a more sensitive parameter to the lower end of the molecular weight distribution, these results would suggest that chain scission is taking place followed by cross linking of the low molecular weight chains of the cast POSS-PCU. An increase in ATR-FTIR peak intensity at 1095 cm^−1^ would reinforce these findings because ∼1100 cm^−1^ is the region where we would expect to find the ether peak as a result of cross linking. However, it is difficult to assign this peak with any certainty as the Si-O-Si of POSS is also expected in this region.

There is no evidence to suggest any cross-linking is taking place in the POSS-PCL samples. GPC data showed significant reductions in *M*_n_ and *M*_w_ for both the cast and porous samples suggesting severe degradation of samples. These findings were reinforced by the ATR-FTIR results, which found reductions in peaks at 1730, 1540, 1240, and 1165 cm^−1^ indicating chain scission in both the hard and soft segments.

The effect of polymer degradation was seen in the mechanical testing of the samples. The UTS and Young’s modulus decreased significantly for the casted samples of both POSS-PCU and POSS-PCL. The porous samples did not display any significant changes in their mechanical properties. This is probably due to the highly porous nature of the samples meaning that the true cross sectional area on which force was applied was considerably lower than the measured area resulting in lower than expected values. The force range on which the samples were examined may not be large enough to determine any detrimental effects on the polymer chains.

It should be noted, however, that the polymer samples were exposed to gamma irradiation for a continuous 10 h at a dose of 28.4 kGy. Alternative protocols for gamma irradiation may influence the materials differently, for example, higher doses at shorter exposure time periods.

## Conclusion

Sterilizing POSS-modified polyurethanes via EtOH resulted in no observable damage but the sterilization process was inefficient. On the other hand, both autoclaving and gamma irradiation resulted in effective sterilization of samples; although the high temperatures involved in autoclaving resulted in the structural collapse of the biodegradable POSS-PCL samples, the nondegradable POSS-PCU withstood autoclaving well. Gamma irradiation however led to mild degradation of both POSS-PCU and POSS-PCL samples and had a cytotoxic effect on EPC culture. The results indicate that autoclaving is the optimal sterilization procedure for POSS-PCU; however, more suitable methods to sterilize biodegradable materials are needed. Tissue engineering scaffolds are purposefully designed to degrade over time; however, their fragile nature also makes them unsuitable for the standard forms of sterilization. Therefore, new methods capable of sterilizing without affecting them chemically, mechanically, or morphologically are needed for *in vivo* use of biodegradable scaffolds.
